# A highly expressed mRNA signature for predicting survival in patients with stage I/II non-small-cell lung cancer after operation

**DOI:** 10.1038/s41598-021-85246-x

**Published:** 2021-03-12

**Authors:** Nan Ma, Lu Si, Meiling Yang, Meihua Li, Zhiyi He

**Affiliations:** Department of Respiratory Medicine, The First Affiliated Hospital of GuangXi Medical University, Nanning, 530021 GuangXi People’s Republic of China

**Keywords:** Cancer genomics, Gene expression, Genetic markers

## Abstract

There is an urgent need to identify novel biomarkers that predict the prognosis of patients with NSCLC. In this study,we aim to find out mRNA signature closely related to the prognosis of NSCLC by new algorithm of bioinformatics. Identification of highly expressed mRNA in stage I/II patients with NSCLC was performed with the “Limma” package of R software. Survival analysis of patients with different mRNA expression levels was subsequently calculated by Cox regression analysis, and a multi-RNA signature was obtained by using the training set. Kaplan–Meier estimator, log-rank test and receiver operating characteristic (ROC) curves were used to analyse the predictive ability of the multi-RNA signature. RT-PCR used to verify the expression of the multi-RNA signature, and Westernblot used to verify the expression of proteins related to the multi-RNA signature. We identified fifteen survival-related mRNAs in the training set and classified the patients as high risk or low risk. NSCLC patients with low risk scores had longer disease-free survival than patients with high risk scores. The fifteen-mRNA signature was an independent prognostic factor, as shown by the ROC curve. ROC curve also showed that the combined model of the fifteen-mRNA signature and tumour stage had higher precision than stage alone. The expression of fifteen mRNAs and related proteins were higher in stage II NSCLC than in stage I NSCLC. Multi-gene expression profiles provide a moderate prognostic tool for NSCLC patients with stage I/II disease.

## Introduction

Non-small-cell lung cancer (NSCLC) is a disease with high morbidity and mortality rates, accounting for approximately 85% of lung cancer cases^1,2^. Although Surgery plays a pivotal role in treating NSCLC, 5-year survival rates of NSCLC after surgical resection are commonly accepted to be 60% to 80% for stage I and 30% to 50% for stage II^3^. The prognosis is more favourable in localized or limited advanced stages. The risk of recurrence peaks within the first 2 years after the operation^4^. Most postoperative recurrences are found during routine follow-up when patients are asymptomatic. Hence, it is urgent to explore effective NSCLC prognostic biomarkers to help optimize clinical management and ultimately further improve clinical outcome.

Gene expression can be used as a surrogate measurement of cancer disease phenotype^5,6^. Multiple gene signatures are found by using bioinformatics technology, and considered to have an intimate association with the prognosis of NSCLC^7–9^. High expression of some genes is closely related to cancer progression, which can be used to determine patient prognosis^10–12^. As such, numerous highly expressed genes with various inherent and acquired genetic alterations have been shown to influence NSCLC prognosis^1,7,8,13,14^. Screening tumor markers based on bioinformatics technology is a hot spot in current research, so the aim of this study is try to use a new algorithm to screen highly expressed mRNAs may have significant prognostic value in the recurrence of patients with NSCLC.

Microarray technology and bioinformatic analysis have been increasingly regarded as useful methods to identify biomarkers as diagnostic and prognostic tools^15,16^. With the help of gene expression databases such as Gene Expression Omnibus (GEO), it is easy to obtain abundant expression data for NSCLC. It is very helpful to analyse NSCLC at the genetic level. These resources have improved our ability analyse NSCLC at the genetic level. Data on individual patients' mRNA profiles and clinical information can be obtained from Affymetrix human genome U133 plus 2.0 array^17,18^ and GEO data sets, after screening the data of mRNA, stage I and II patients are complete and suitable for analysis using bioinformatics, so we performed this research to find a multi-RNA prognostic signature of highly expressed mRNAs for predicting relapse in stage I/II patients with NSCLC after surgery by analysing the GEO data.

## Results

### Identify survival-related mRNA in the training set

**Figure 1:**
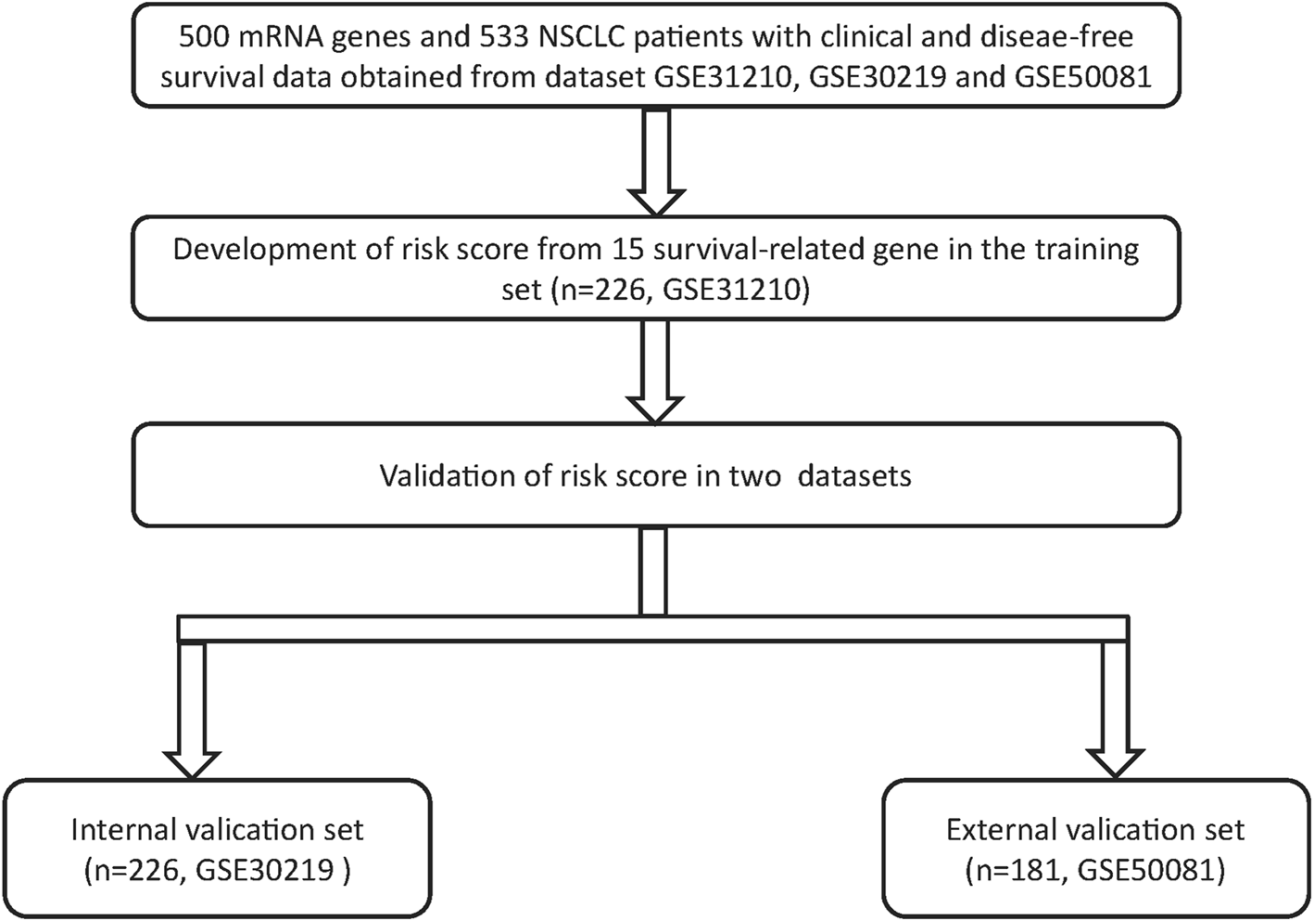
Development and validation of the fifteen-mRNA signature shown as study flow.

**Figure 2:**
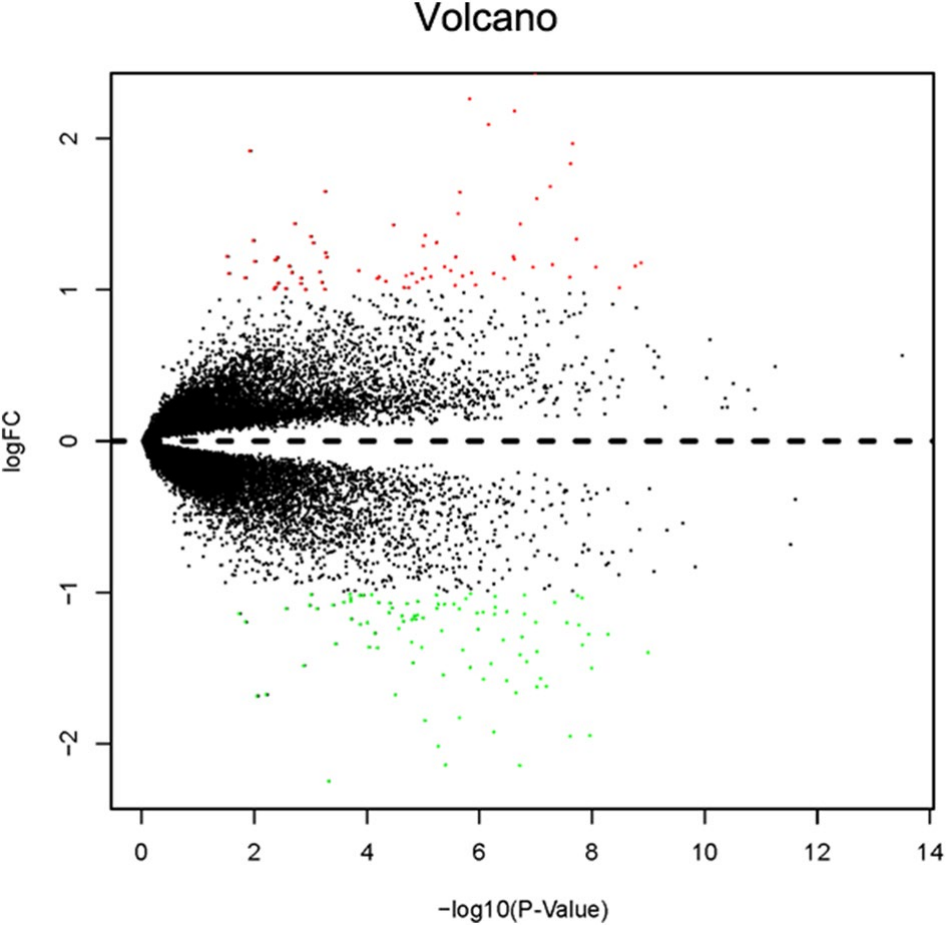
Volcano plot of mRNA expression in the training set (n = 226, GSE31210). The red points in the volcano plot represents the statistically significant highly expressed mRNAs, and the green points represent mRNAs with significantly low expression.

**Figure 3:**
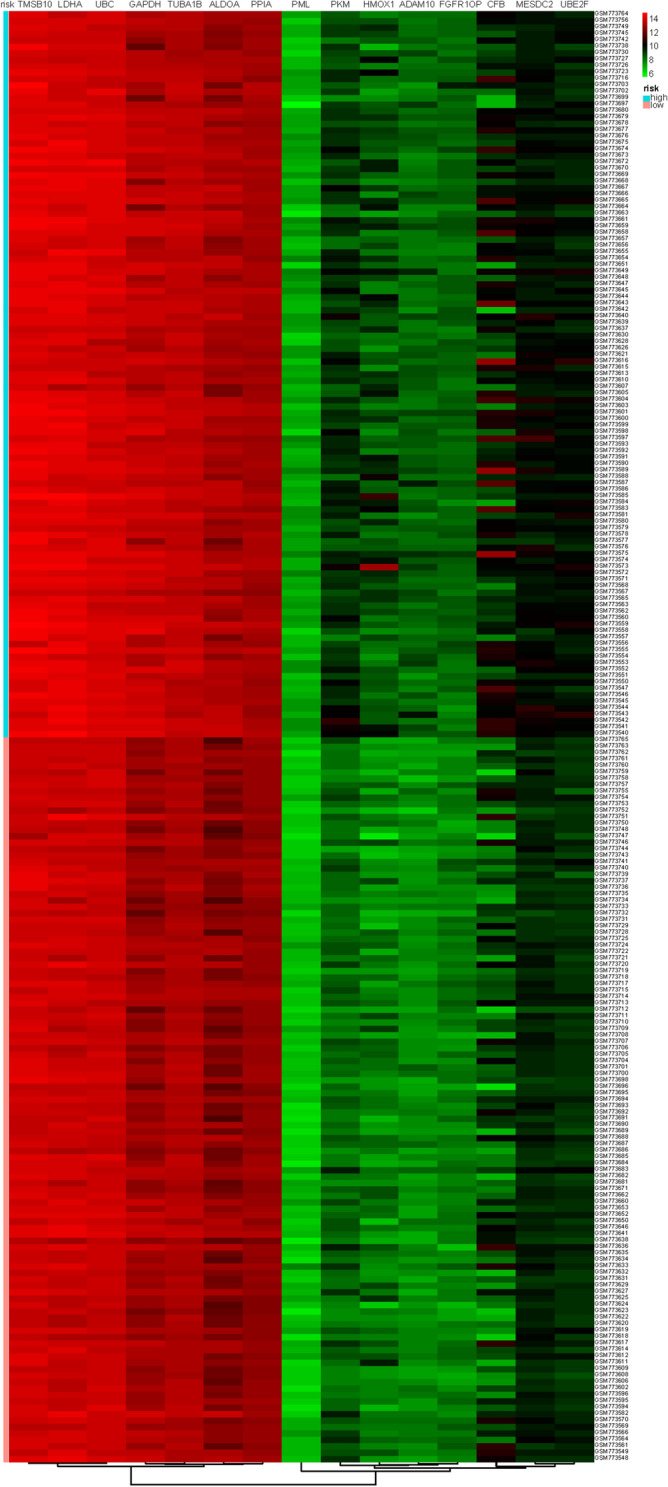
Heatmap of the fifteen-mRNA signature and risk scores in the training set(n = 226, GSE31210). The “pheatmap” package of R software (version 3.5.1) was used to generate the heatmap.

The fifteen-mRNA signature of highly expressed mRNAs associated with survival was developed and validated as shown in Fig. 1. We identified highly expressed mRNAs from GSE31210 by using microarray data. Differentially expressed mRNAs were selected by volcano plot filtering (fold change ≥ 1 and *P*-value ≤ 0.05, Fig. 2). The relationship between RFS, survival state and high expression genes in NSCLC patients was performed using univariate Cox regression analysis in the training set, "risk score" of highly expressed genes in NSCLC prognosis was calculated. The higher the risk score, the greater the correlation between mRNA and RFS. According to the results, the fifteen-mRNA signature was significantly associated with RFS (n = 226, GSE31210; Table 1). UBE2F, TMSB10 and GAPDH were negative coefficients, indicating that patients with higher levels of expression had better outcomes than patients with lower levels of expression. Twelve mRNAs (UBC, TUBA1B, PPIA, PML, PKM, MESDC2, LDHA, HMOX1, FGFR1OP, CFB, ALDOA and ADAM10) were considered to be positive coefficients, so high levels of these mRNAs were associated with worse outcomes. Heatmap visualized distributions of fifteen-mRNA and risk scores in the training set and two independent GEO cohorts (Fig. 3).

**Table 1:** The characteristics of the fifteen-mRNA signature related to RFS in the training set (n = 226, GSE31210).

Gene symbol	HR	Coefficients	P-value	Putative function
ADAM10	1.002(0.9994–1.005)	0.00220	< 0.01	Risky
ALDOA	1(1–1)	0.00018	< 0.01	Risky
CFB	1(0.9999–1)	0.00016	< 0.01	Risky
FGFR1OP	1.003(0.9989–1.007)	0.00283	< 0.01	Risky
GAPDH	0.9999(0.9997–1)	− 0.00013	< 0.01	Protective
HMOX1	1.001(1.0002–1.002)	0.00084	< 0.01	Risky
LDHA	1(0.9999–1)	0.00003	< 0.01	Risky
MESDC2	1.001(0.9994–1.002)	0.00060	< 0.01	Risky
PKM	1(0.9985–1.002)	0.00003	< 0.01	Risky
PML	1.006(0.9968–1.016)	0.00622	< 0.01	Risky
PPIA	1(0.9997–1.001)	0.00014	< 0.01	Risky
TMSB10	0.9999(0.9998–1)	− 0.00007	< 0.01	Protective
TUBA1B	1(0.9999–1)	0.00017	< 0.01	Risky
UBC	1(0.9999–1)	0.00010	< 0.01	Risky
UBE2F	0.9993(0.9978–1.001)	− 0.00065	< 0.01	Protective

### Survival analyses between low-risk and high-risk groups

**Figure 4:**
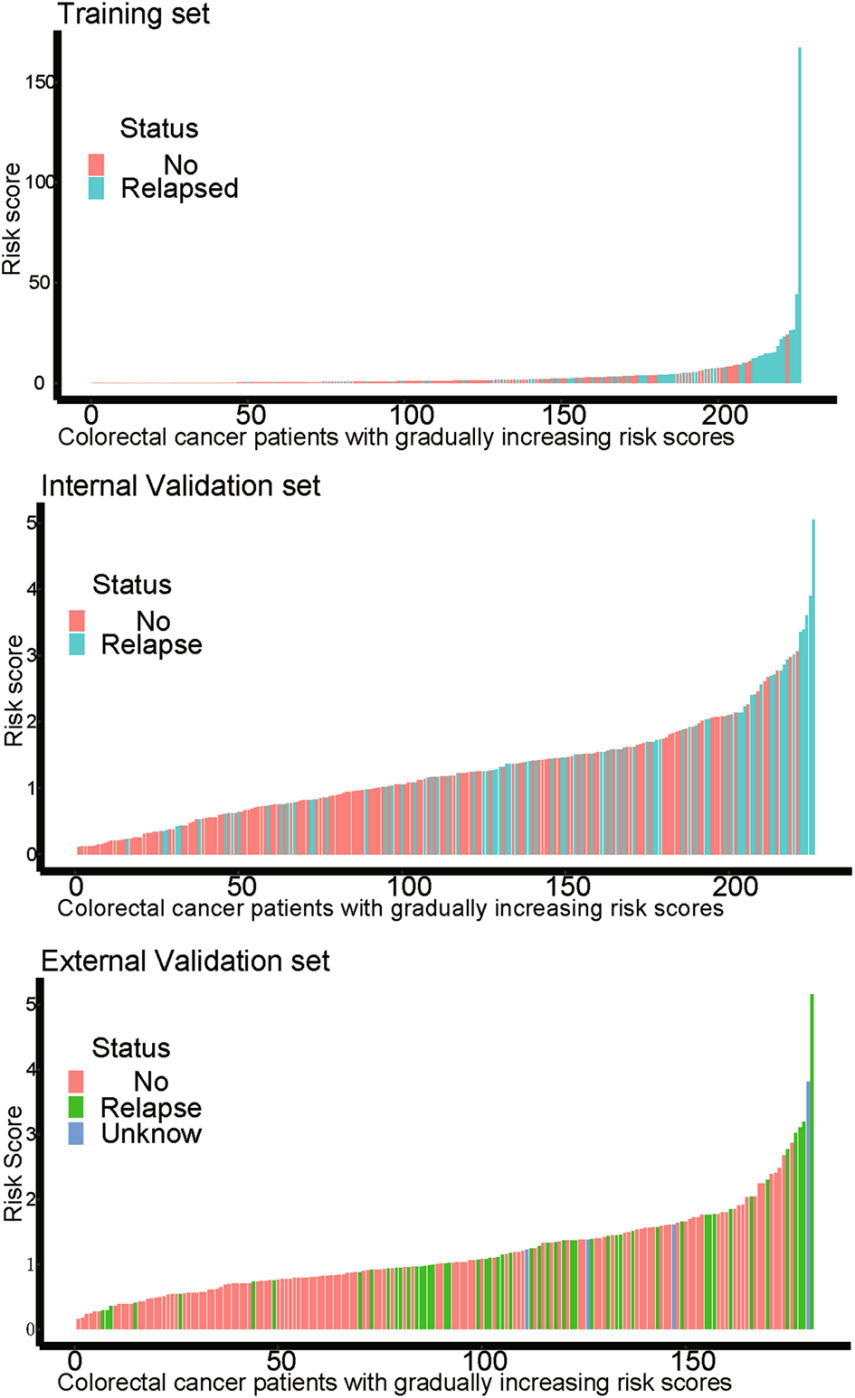
The distributions of RFS status and risk score in the training (n = 226, GSE31210) internal validation (n = 226, GSE30219) and external validation (n = 181, GSE50081) sets. The results showed that patients with recurrent NSCLC had a high risk score. Abbreviation: RFS, disease-free survival.

**Figure 5:**
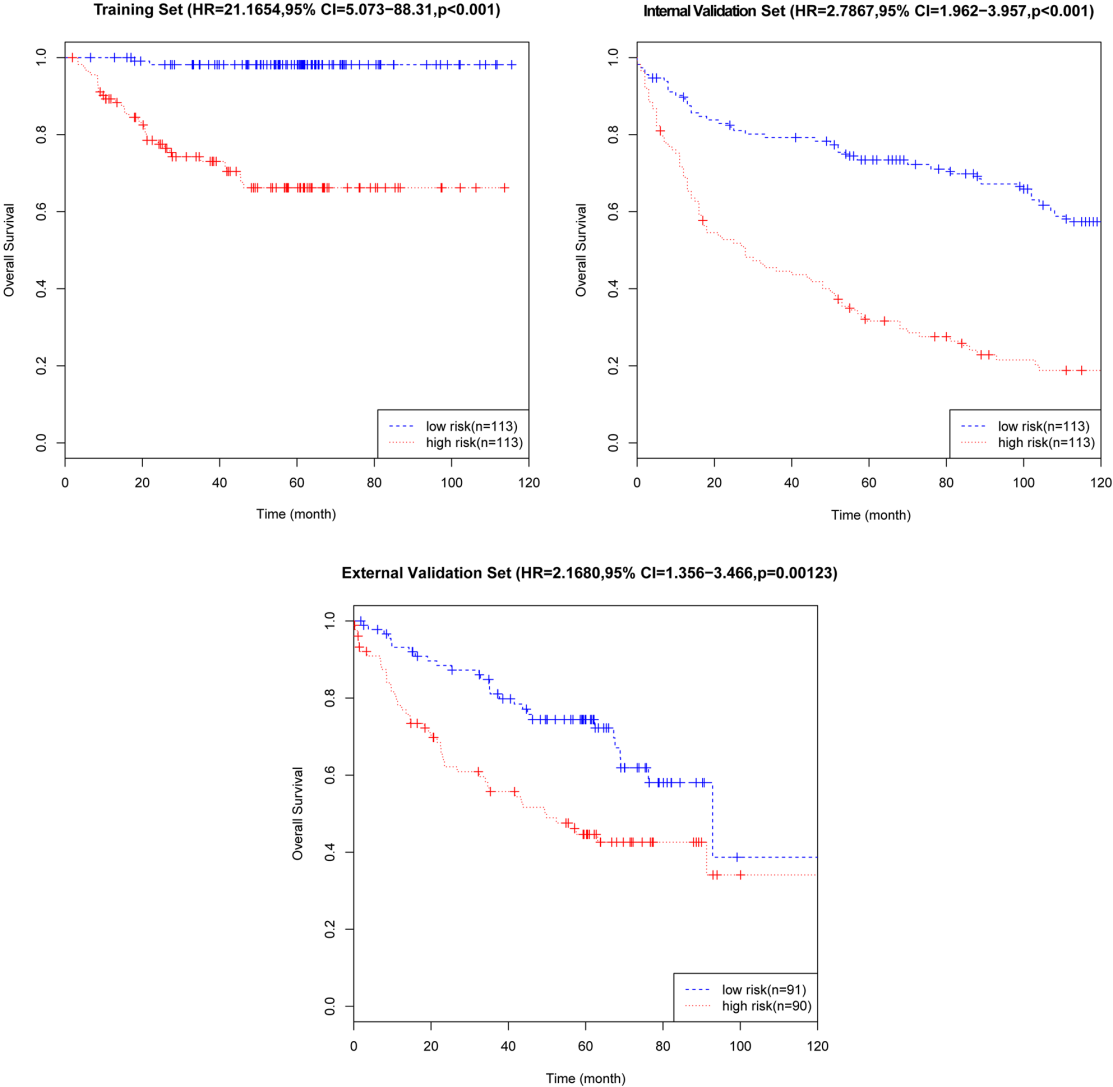
Kaplan–Meier curves of disease-free survival on the basis of the fifteen-mRNA signature in the training, internal validation, and external validation sets. As the result denmonstrated lower scores were associated with longer RFS, and higher scores were associated with shorter RFS in each set (*P* < 0.05).

On the basis of the expression of mRNAs and their regression coefficients in the multivariate Cox model, we determined individual patient risk scores according to the fifteen-mRNA in the training set, internal validation set and external validation set. As the median value was used as the cutoff value, NSCLC patients in each set were classified into the low-risk group or the high-risk group. Figure 4 shows the distributions of risk score and RFS status in each set, it demonstrated NSCLC patients who had high risk scores had a higher risk of relapse after surgery. The clinical characteristics of low-risk group and high-risk group patients in these three sets are shown in Table 2. As our result, the clinical characteristics of the external independent variables (age, sex, stage) between the low-risk group and the high-risk group were not significantly different. RFS analyses were performed by log-rank test to determine the differences between high-risk and low-risk groups in these three sets (Fig. 5 and Table 3); lower scores were associated with longer RFS, and higher scores were associated with shorter RFS in each set (*P* < 0.05). These results suggest that these fifteen-mRNA can distinguish NSCLC patients with different prognosis, and can be used in subsequent studies.

**Table 2:** Clinical characteristics of NSCLC patients on the basis of the fifteen-mRNA signature in the training (n = 226, GSE31210) internal validation (n = 226, GSE30219) and external validation (n = 181, GSE50081) sets.

Characteristics	Training Set(n = 226, GSE31210)			Internal Validation Set(n = 226, GSE30219)			External Validation Set (n = 181, GSE50081)		
	High-risk (n = 113)	Low-risk (n = 113)	*P-*value	High-risk (n = 113)	Low-risk (n = 113)	*P-*value	High-risk (n = 90)	Low-risk (n = 91)	*P-*value
**Age (years)**									
< 60	51	45	0.5	38	50	0.133	12	15	0.677
≥ 60	62	68		75	63		78	76	
**Gender**									
Male	61	44	0.03	104	88	0.005	41	41	1
Female	52	69		9	25		49	49	
**Stage**									
I	61	107	< 0.01	71	90	< 0.01	64	63	1
II	52	6		42	23		26	27	

The clinical characteristics of the external independent variables (age, sex, stage) between the low-risk group and the high-risk group were not significantly different.

**Table 3:** RFS analyses by the log-rank test according to the fifteen-mRNA signature in each set. Our result shown lower scores were associated with longer RFS, and higher scores were associated with shorter RFS in each set (*P* < 0.05).

Datasets	Risk group (n)	Recurrence-free survival				
		1-year	3-year	5-year	HR (95% CI)	*P*-value
Training set (n = 226, GSE31210)	High-risk (n = 113)	83.19%	53.98%	29.20%	21.17 (5.073–88.31)	< 0.001
	Low-risk (n = 113)	99.12%	84.07%	54.87%		
Internal validation set (n = 226, GSE30219)	High-risk (n = 113)	68.14%	43.36%	28.32%	2.787 (1.962–3.957)	< 0.001
	Low-risk (n = 113)	86.73%	76.11%	61.95%		
External validation set (n = 181, GSE50081)	High-risk (n = 90)	74.44%	46.67%	31.11%	2.168 (1.356–3.466)	0.001
	Low-risk (n = 91)	89.01%	71.43%	43.96%		

### Multivariate Cox regression analysis of the fifteen-mRNA signature and clinical information in each set

The relationship of the fifteen-mRNA signature, clinical information (sex, age, stage) and RFS in each set was analysed by multivariate Cox regression analysis (Table 4). As our data showed, the fifteen-mRNA signature was significantly related to the RFS as well as clinical characteristic in these datasets of NSCLC patients (all *P* < 0.05).

**Table 4:** The relationship of the fifteen-mRNA signature, clinical information (sex, age, stage) and RFS in each set analysed by multivariate Cox regression.

Datasets	Variable	Recurrence-free surviva	
		HR (95% CI)	P-value
Training Set (n = 226, GSE31210)	Fifteen-mRNA classifier (high- vs low-risk)	17.98067 (4.1336–78.214)	0.0001
	Age (≥ 60 years vs < 60 years)	1.04389 (0.9956–1.095)	0.0754
	Gender (female vs male)	1.05406 (0.5397–2.059)	0.8775
	Stage	1.78139 (0.8990–3.530)	0.098
Internal Validation Set (n = 226, GSE30219)	Fifteen-mRNA classifier (high- vs low-risk)	2.26383 (1.5823–3.239)	< 0.05
	Age (≥ 60 years vs < 60 years)	1.04535 (1.0280–1.063)	< 0.05
	Gender (female vs male)	1.30206 (0.7287–2.326)	0.3728
	Stage	1.77690 (1.2508–2.524)	0.001
External Validation Set (n = 181,GSE50081)	Fifteen-mRNA classifier (high- vs low-risk)	2.11150 (1.3189–3.381)	0.003
	Age (≥ 60 years vs < 60 years)	1.01390 (0.9897–1.0390)	0.262
	Gender (female vs male)	0.50440 (0.3097–0.822)	0.006
	Stage	1.79920 (1.1147–2.904)	0.017

As our data showed, the fifteen-mRNA signature was significantly related to the RFS as well as clinical characteristic in these datasets of NSCLC patients (all *P* < 0.05).

### ROC analysis of the fifteen-mRNA signature and stage in each set

**Figure 6:**
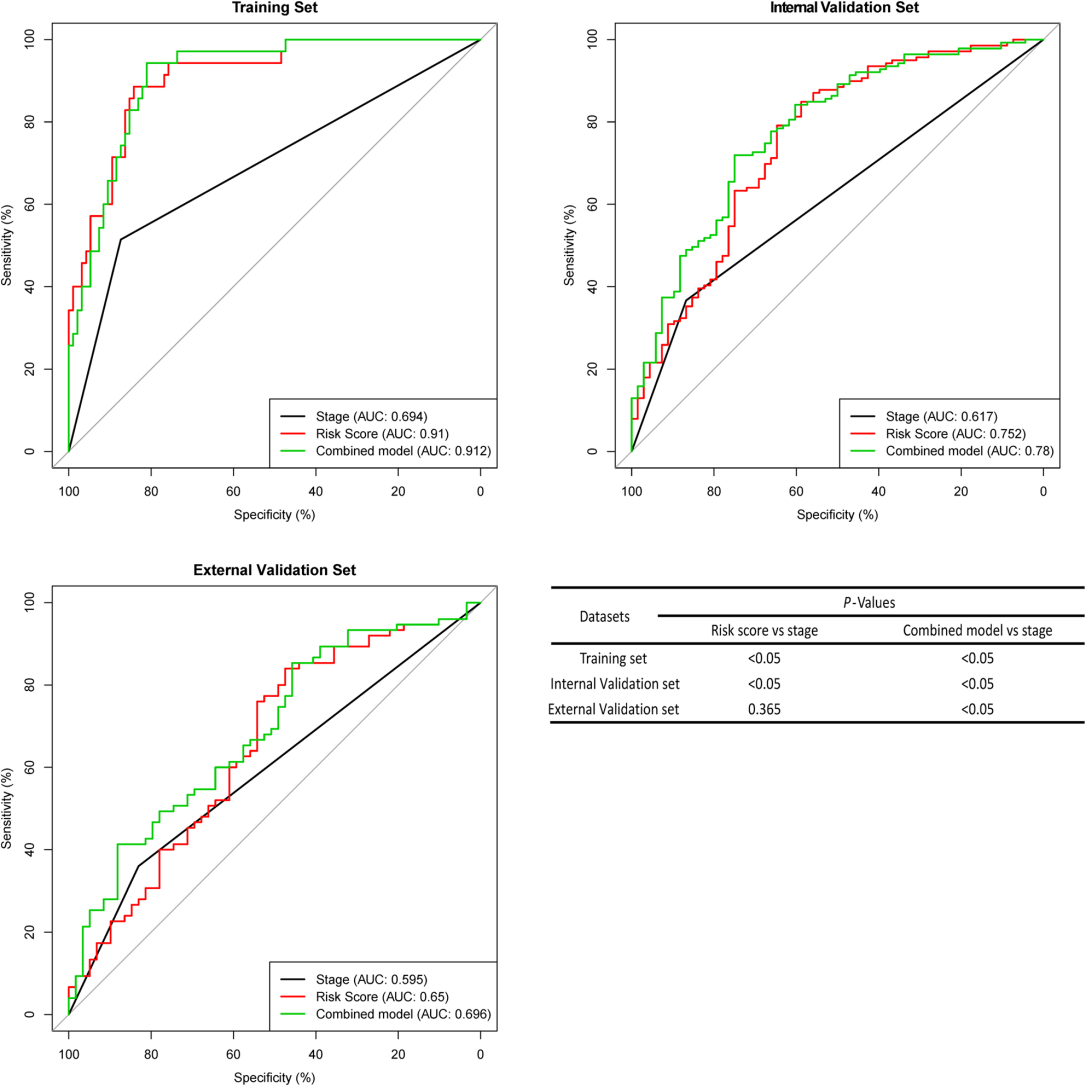
ROC curves of the combined model of the fifteen-mRNA signature and stage, the fifteen-mRNA signature and stage alone for each set. The AUC of the fifteen-mRNA signature was higher than that of stage alone in the training (*P* < 0.05) and internal validation sets (*P* < 0.05). The combined model’s (fifteen-mRNA signature and tumour stage) AUC was higher than that of stage alone in each set (*P* < 0.05). Abbreviation: AUC, area under the curve.

The area under the curve (AUC) of the ROC curve was used to analyse the RFS of fifteen-mRNA signatures and stages in each set (Fig. 6). As the figure shows, the AUC of the fifteen-mRNA signature was higher than that of stage alone in the training (*P* < 0.05) and internal validation sets (*P* < 0.05). The combined model’s (fifteen-mRNA signature and tumour stage) AUC was higher than that of stage alone in each set (*P* < 0.05). It suggests that fifteen-mRNA signatures have good predictive power to the prognosis of NSCLC patients.

### Comparison of RFS in the combined set (training set and internal validation set)

**Figure 7:**
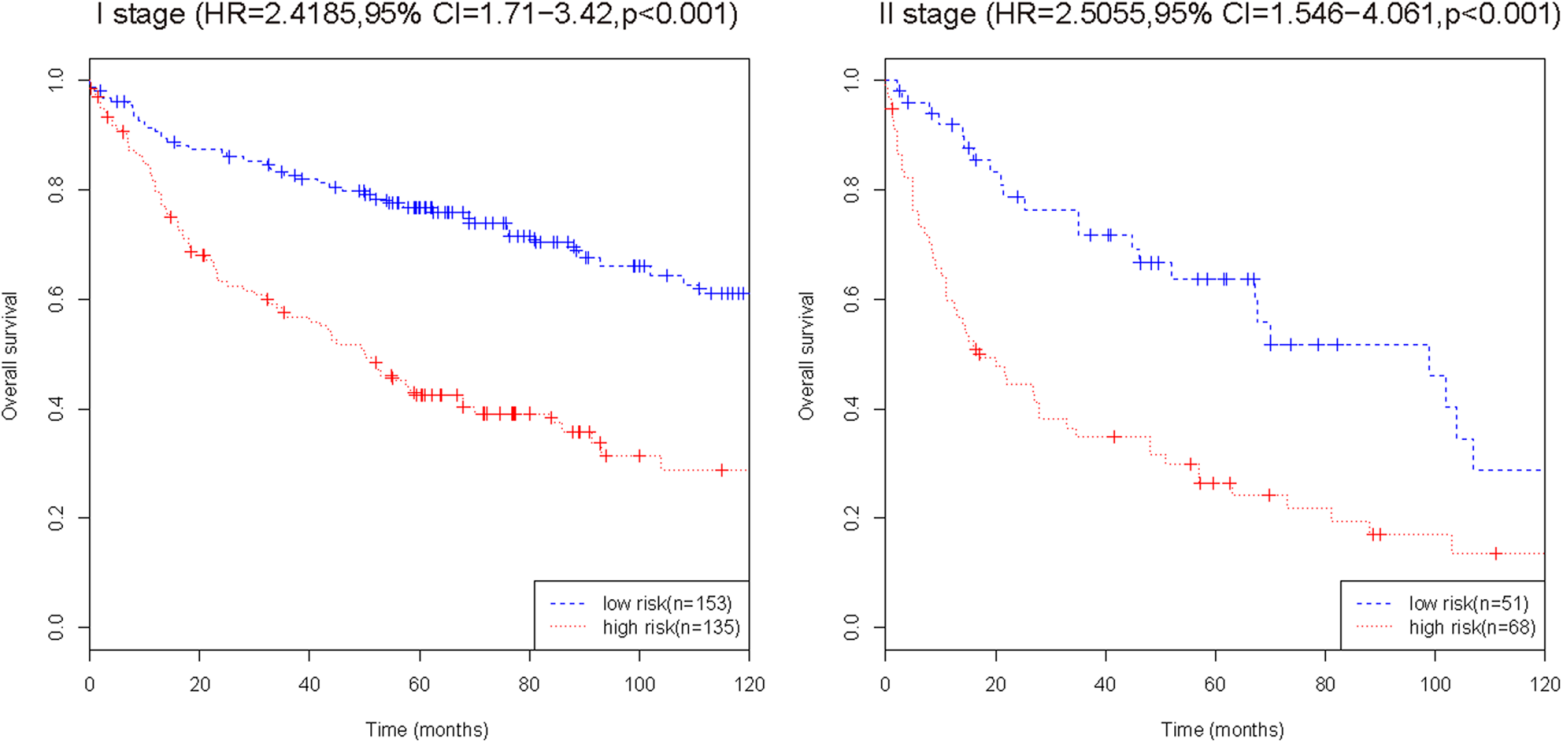
Kaplan–Meier curve analysis of RFS according to the fifteen-mRNA signature for stage I/II patients in the combined training set and internal validation set. The Kaplan–Meier curve further showed that the RFS of the high-risk group was significantly shorter than that of the low-risk group (*P* < 0.001).

To further verify the efficacy of this fifteen-mRNA signature, we merged the training set and internal validation set into a combined set (n = 407, GSE31210 and GSE30219) and compared the RFS of the low-risk group (n = 204) and high-risk group (n = 203). The results show that the RFS of the high-risk group was significantly shorter than that of the low-risk group (*P* < 0.001, Table 5). Survival analysis of clinical information (sex, age, stage) and mRNA in the combined set using multivariate Cox regression. The results showed a significant correlation between our mRNA signature and RFS (HR = 2.30743, 95% CI = 1.7407–3.059, *P* < 0.001; Table 6). The Kaplan–Meier curve further showed that the RFS of the high-risk group was significantly shorter than that of the low-risk group (*P* < 0.001, Fig. 7). The results in combined set are consistent with our previous analysis.

**Table 5:** Comparison of RFS in patients with NSCLC based on the fifteen-mRNA signature in the combined training set and the validation set (n = 407, GSE30219 and GSE50081).

Set	Risk group (n)	Recurrence-free surviva				
		1-year	3-year	5-year	HR (95% CI)	*P*-value
The combined set (n = 407)	High-risk (n = 203)	70.94%	45.32%	29.56%	2.558(1.932–3.39)	< 0.001
	Low-risk (n = 204)	87.75%	74.02%	53.92%		

The results show that the RFS of the high-risk group was significantly shorter than that of the low-risk group (*P* < 0.001).

**Table 6:** Predictive value of the fifteen-mRNA signature, sex, age, stage, and survival in the combined set (n = 407, GSE30219 and GSE31210) analysed by multivariate Cox regression.

Set	Risk group (n)	Recurrence-free survival	
		HR (95% CI)	*P*-value
The combined set (n = 407)	Fifteen-mRNA classifier (high- vs low-risk)	2.30743(1.7407–3.059)	< 0.001
	Age (≥ 60 years vs < 60 years)	1.03088(1.0176–1.044)	< 0.001
	Gender (female vs male)	1.01920(0.7578–1.371)	0.9
	Stage	1.78282(1.3463–2.361)	< 0.001

The results showed a significant correlation between our mRNA signature and RFS (HR = 2.30743, 95% CI = 1.7407–3.059, *P* < 0.001).

### GO and KEGG functional enrichment analysis

**Figure 8:**
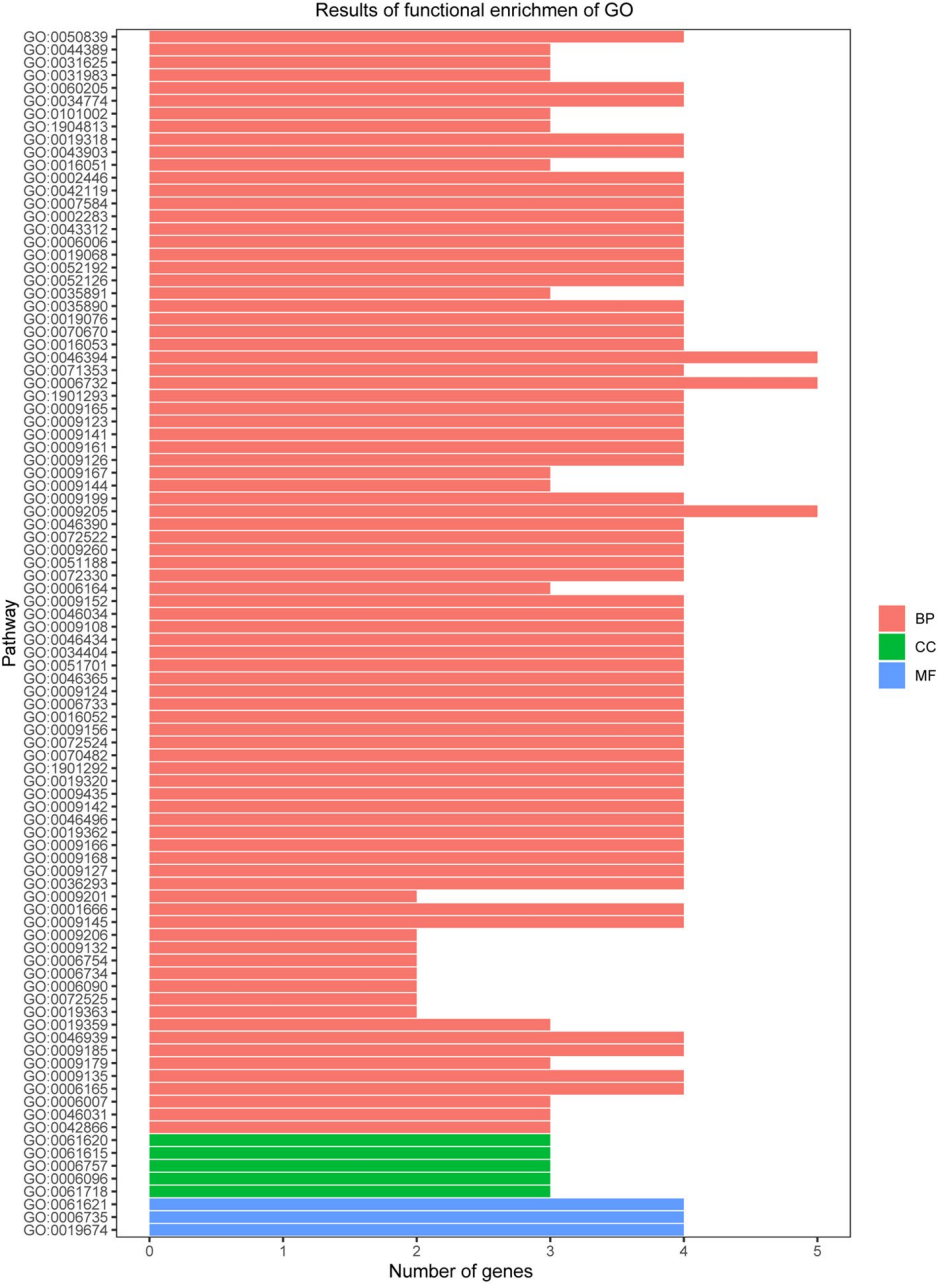
Functional enrichment analysis by GO category (BP: biological process; CC: cell component; MF: molecular function). The GO terms mainly fit into three functional categories: carboxylic acid biosynthetic process (GO: 0,046,394), coenzyme metabolic process (GO: 0,006,732), and purine ribonucleoside triphosphate metabolic process (GO: 0,009,205).

**Figure 9:**
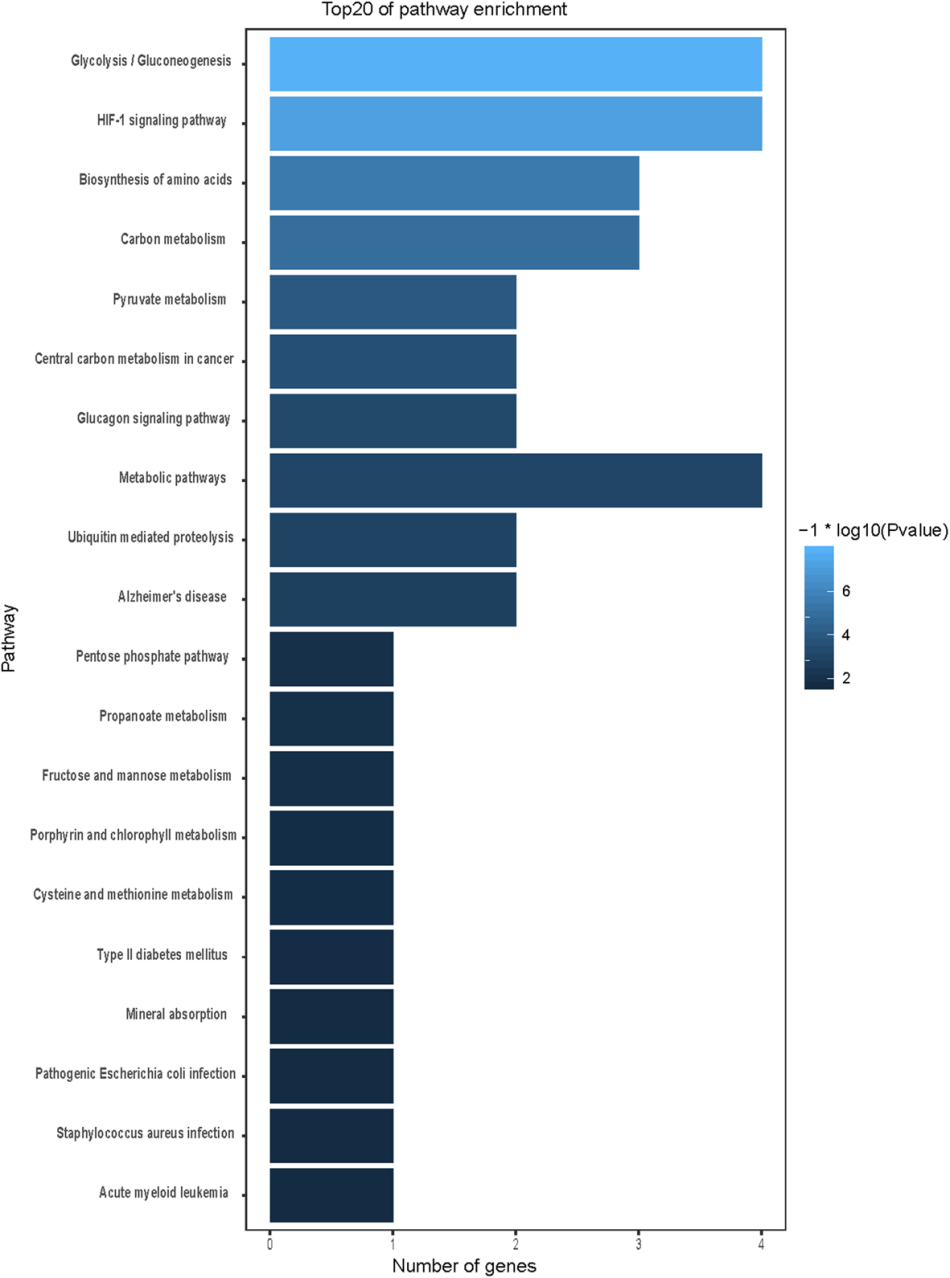
Calculated results of KEGG functional enrichment. Glycolysis/gluconeogenesis (KEGG: 00010) and HIF-1 signalling pathway (KEGG: 04,066) were the main KEGG pathways involved.

We used GO and KEGG enrichment to identify the biological functions and signalling pathways of fifteen-mRNA signature. The results showed that the fifteen-mRNA signature was significantly associated with 94 GO terms (Fig. 8) and 20 KEGG pathways (Fig. 9). The GO terms mainly fit into three functional categories: carboxylic acid biosynthetic process (GO: 0,046,394), coenzyme metabolic process (GO: 0,006,732), and purine ribonucleoside triphosphate metabolic process (GO: 0,009,205). Glycolysis/gluconeogenesis (KEGG: 00010) and HIF-1 signalling pathway (KEGG: 04,066) were the main KEGG pathways involved.

### Protein–protein interaction analysis and mRNA expression validation

**Figure 10:**
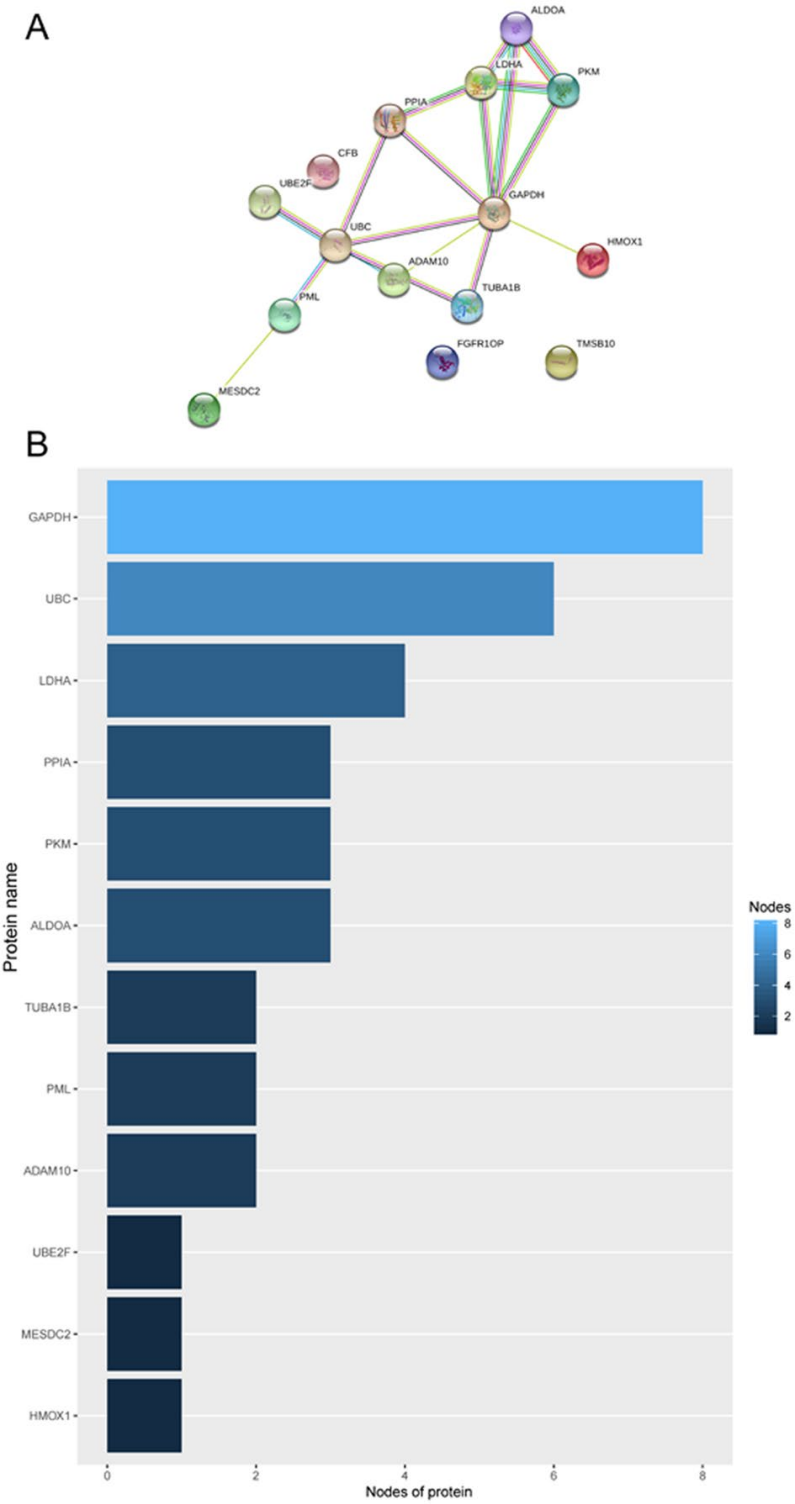
Protein–protein interaction network (**A**) and nodes (**B**) of proteins encoded by the fifteen mRNAs in the signature.

**Figure 11:**
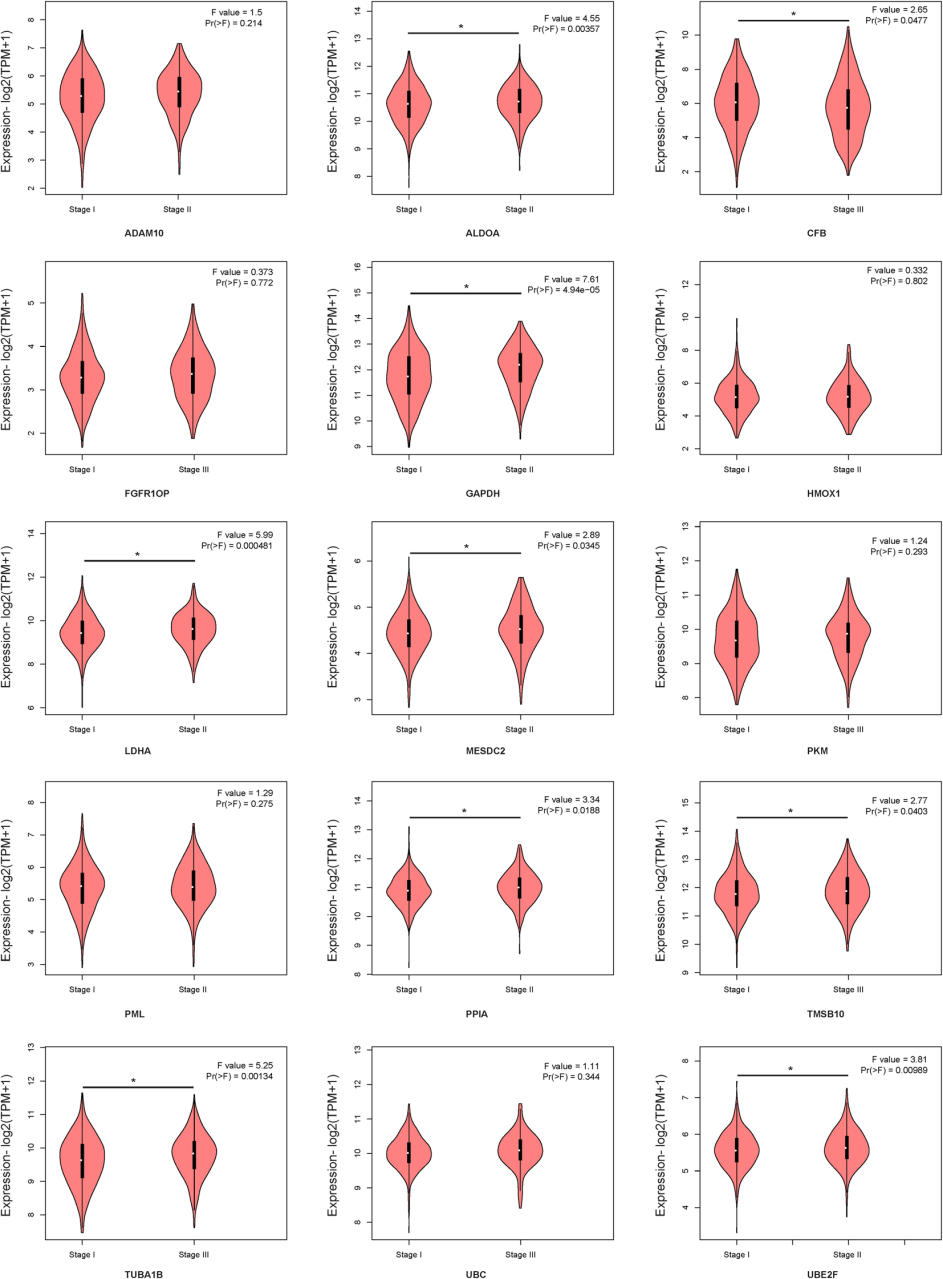
GEPIA online software verified the expression of the fifteen-mRNA signature in stage I/II patients with lung adenocarcinoma and lung squamous cell carcinoma.

STRING online software was used to analyse the interaction between proteins encoded by the fifteen mRNAs (Fig. 10A), and key genes were analysed according to the number of nodes using R software (version 3.5.1). Nodes were mainly interrelated with GAPDH and UBC, so these two proteins were speculated to be the key proteins in this protein–protein interaction network (Fig. 10B). GEPIA online software was used to verify the expression of the fifteen mRNAs in stage I and II patients with lung adenocarcinoma and lung squamous cell carcinoma. The expression of ALDOA, CFB, GAPDH, LDHA, MESDC2, PPIA, TMSB10, TUBA1B, and UBE2F was higher in stage II patients than in stage I patients (Fig. 11, *P* < 0.05).

### The fifteen-mRNA mRNA expression in patients with NSCLC

**Figure 12:**
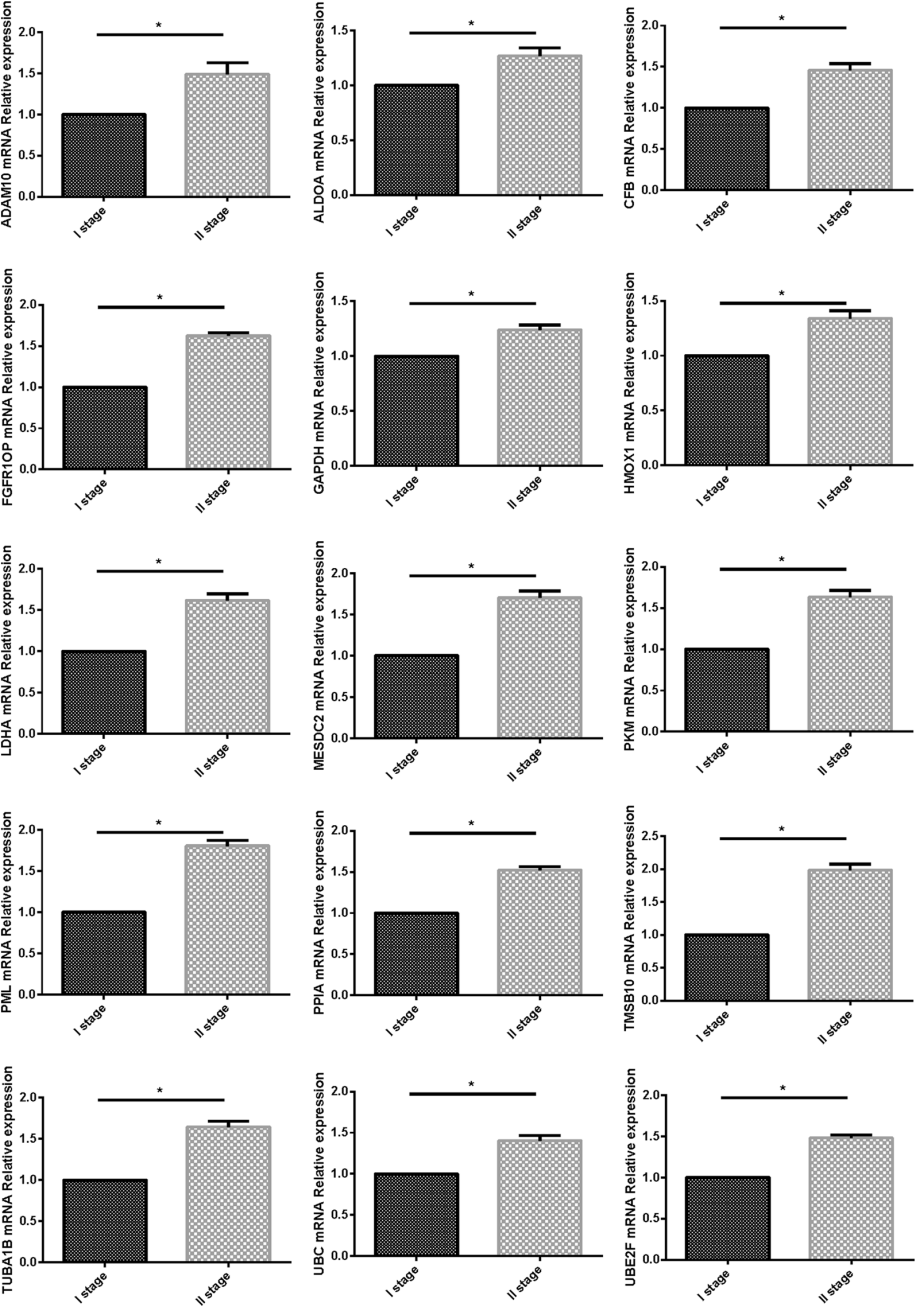
The expression of 15 mRNAs detected by RT-PCR. 15 mRNAs was significantly higher in stage II NSCLC than in stage I ( *P* < 0.05).

We used PCR to verify the expression of the fifteen-mRNA in lung cancer tissues of NSCLC patients, and the results showed that the fifteen-mRNA was significantly higher in stage II NSCLC than in stage I (Fig. 12, *P* < 0.05).

### The proteins related to fifteen-mRNA mRNA expression in patients with NSCLC

**Figure 13:**
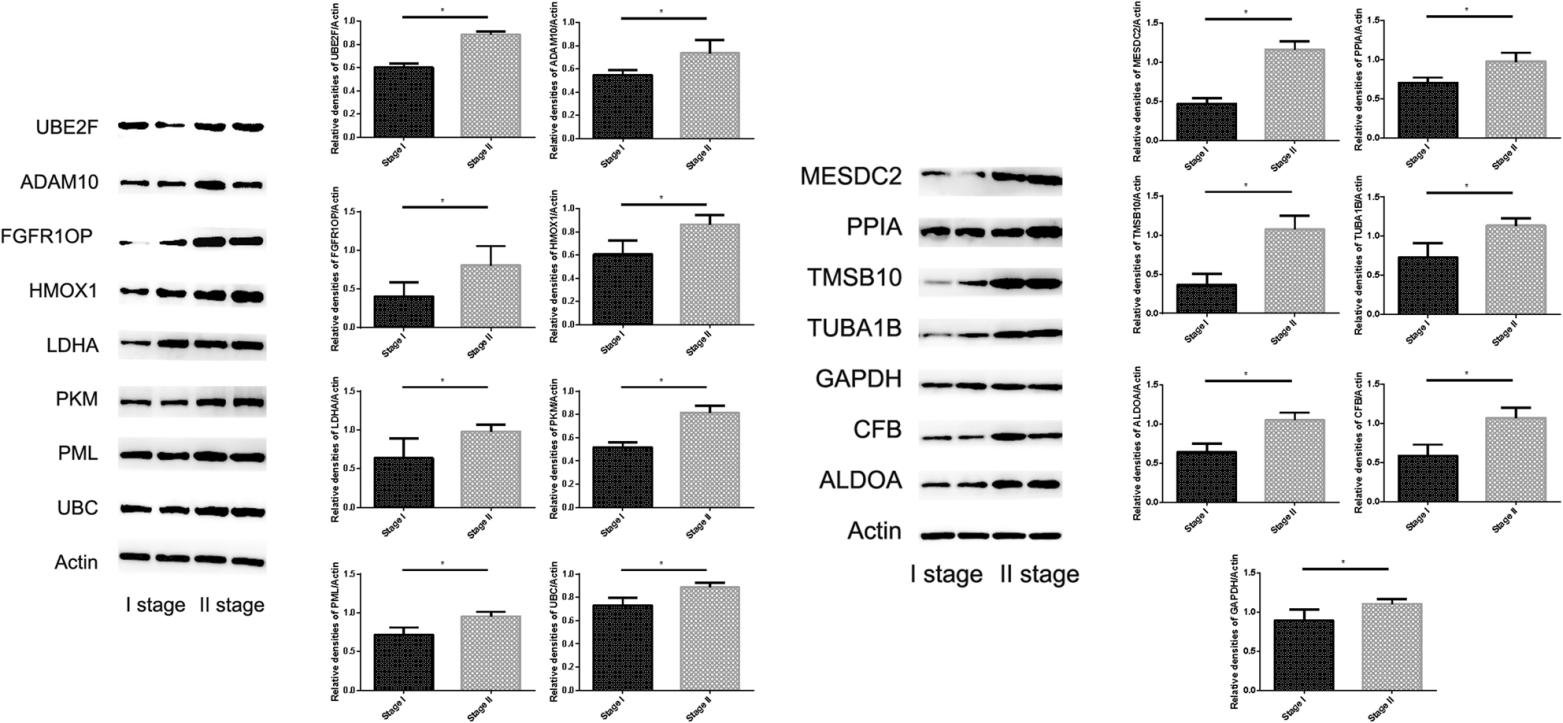
The expression of 15 mRNAs related protein in tumor tissue detected by Westernblot. These 15 mRNAs related protein was significantly higher in stage II NSCLC than in stage I ( *P* < 0.05).

We used westernblot to verify the expression of proteins related to the fifteen-mRNA in lung cancer tissues of NSCLC patients, and the results showed that these proteins was significantly higher in stage II NSCLC than in stage I (Fig. 13, *P* < 0.05).

## Discussion

In general, the TNM system is a widely used staging system among clinicians^19,20^, and TNM staging is essential for evaluating outcomes in clinical practice and for providing some indication of prognosis for survival^21^. Unfortunately, current methods of classification and staging for NSCLC are not completely reliable or sufficiently precise^22–24^. The progression and prognosis of tumours are related to the high expression of some genes^25,26^. The aim of this study was to characterize tumour recurrence and analyse genes related to the increased risk of recurrence in NSCLC. Bioinformatics analysis is currently considered to be an important tool for identifying tumour biomarkers. We profiled NSCLC mRNA by analysing the microarray data of the Affymetrix human genome U133 plus 2.0 array downloaded from GEO. High mRNA expression in stage I/II NSCLC was determined by the “Limma” package in the training set (GSE31210). Univariate Cox proportional hazards regression was used to analyse relationship between high expression genes and patient’s survival time and prognosis in NSCLC, the "risk score" of highly expressed genes in NSCLC prognosis was calculated, In this algorithm, the high risk socre of mRNA related with the poor prognosis of NSCLC. We selected 15 mRNAs with the highest risk score through this algorithm, and peculate that these 15 mRNAs are closely related to the prognosis of NSCLC. In order to study the predictive ability of these 15 mRNAs, we verify them in the training set and two independent GEO cohorts (GSE30219 and GSE50081), and the mRNA signature showed prognostic significance in three cohorts. Many factors, such as sex, age, and stage, are thought to be possible pathogenesis of NSCLC cancer. We analysed NSCLC patient RFS by multivariate Cox regression, and our results showed that the mRNA signature was associated with patient RFS. The mRNA signature performed better than stage alone, and the combined use of RNA and stage performed the best. The combined set, considering the mRNA signature and stage, was significantly associated with patient RFS; in the same stage, our mRNA signature was still significantly associated with patient RFS, and patients with low risk scores had significantly longer RFS. The fifteen-mRNA classifier has a very high HR and a very broad CI in the training set compared with the two other sets, we speculate it may be the instability of the training set. Furthermore, the ROC curve shownd that AUC of the fifteen-mRNA signature was higher than that of stage alone in the training and internal validation sets. The combined model’s (fifteen-mRNA signature and tumour stage) AUC was higher than that of stage alone in each set. Results of ROC curve suggests these fifteen-mRNA signatures as an independent prognostic factor in NSCLC. Finally, our bioinformatics analysis results shown our fifteen-mRNA signature is a novel biomarker with useful applications in predicting NSCLC prognosis.

To determine the biological relationship and signalling pathways among the fifteen mRNAs in the signature, we performed GO and KEGG analyses. Functional categories of the fifteen mRNAs were mainly involved in three GO terms, including the carboxylic acid biosynthetic process (GO: 0,046,394), coenzyme metabolic process (GO: 0,006,732), and purine ribonucleoside triphosphate metabolic process (GO: 0,009,205). All three pathways are considered to be closely related to tumours^27–30^. The main KEGG pathways involved included glycolysis/gluconeogenesis (KEGG: 00010) and the HIF-1 signalling pathway (KEGG: 04,066). Glycolysis is a universal pathway in living cells, and the glycolysis rate is 200 times higher in tumour cells than in normal cells^31^. Previous studies have shown that inhibition of HIF-1 represents a novel approach to cancer therapy^32,33^. We analysed the protein–protein interactions between proteins encoded by fifteen mRNAs. GAPDH and UBC were speculated to be the key proteins in this protein–protein interaction network according to their nodes, it suggest these two mRNA may play the key role of 15 mRNA. The expression of the fifteen mRNAs was validated by GEPIA online software, and the expression levels of ALDOA, CFB, GAPDH, LDHA, MESDC2, PPIA, TMSB10, TUBA1B, and UBE2F were higher in stage II patients than in stage I patients with lung adenocarcinoma and lung squamous cell carcinoma, which was consistent with our previous results in the gene sets.

Furthermore, we verified the expression of mRNA in NSCLC tumor tissues by RT-PCR and confirmed the expression of 15 mRNA related proteins by Westernblot. Our results showed that the expression of 15 mRNA genes was higher in stage II NSCLC than in stage I NSCLC, and the expression of 15 mRNA gene related proteins also showed the same situation, that is, in stage II is higher than in stage I. The fifteen-mRNA signature included twelve risky genes (UBC, TUBA1B, PPIA, PML, PKM, MESDC2, LDHA, HMOX1, FGFR1OP, CFB, ALDOA and ADAM10) and three protective genes (UBE2F, TMSB10 and GAPDH). Previous research showed that high tissue levels of PKM^34,35^, LDHA^36,37^, HMOX1^38^, FGFR1OP^39^, ADAM10^10,40^, ALDOA^41,42^, and GAPDH ^43,44^were correlated with an increased risk of relapse in NSCLC patients. Low expression of UBC inhibits radiostasis and proliferation of NSCLC tumor cells^45^, UBE2F high expression promotes lung cancer cell survival^46^. CFB promote migration and proliferation of Cutaneous Squamous Cell Carcinoma^47^, and overexpression of TMSB10 relate with hepatocellular carcinoma and renal cell carcinoma^48,49^. Although there are no studies on eight of the mRNAs (TUBA1B, UBC, PPIA, PML, MESDC2, CFB, UBE2F, TMSB10) in prognosis of NSCLC, our experimental results shown these 15 mRNAs are involved in the progression of NSCLC, these experimental results provide evidence for the roles of these mRNAs in NSCLC and identify them as biomarkers.

The innovation of this research is identified mRNAs significantly related to RFS of NSCLC with a risk score via univariate Cox analysis, these 15 mRNAs have shown good predictive ability in the training set, internal validation set and external validation set. However, there were several limitations to our study. For example, further experiments are required to verify the clinical value of the signature. Limited by the clinical information of GEO data sets, we cannot identify the resection status of patients with NSCLC. Additionally, our experimental sample size is small, larger clinical trials may lead to more convincing results.

Our findings demonstrate a multiple-mRNA signature closely relate with tumour prognosis in stage I/II patients with NSCLC. It may aid in the development of novel biomarkers of NSCLC and offer new insights into NSCLC prognosis and may provide a new method for analyzing NSCLC based on Cox analysis.

## Methods

### Data of NSCLC

Raw microarray data from all data sets were analysed using the Affymetrix human genome U133 plus 2.0 array (GSE31210, GSE30219 and GSE50081), the mRNA expression data were log2 transformed before statistical analysis, and the median value was used when multiple probes existed for a single target. There was a total of 627 stage I/II patients with NSCLC after excluding patients without Recurrence Free Survival(RFS) or clinical data, including 226 from GSE31210, 226 from GSE30219 and 181 from GSE50081. The 226 patients from GSE31210 were used as a training set, 226 patients from GSE30219 were used as an internal validation set, and 181 patients from GSE50081 were used as the external validation set. The training set was used to optimize the parameters of model, and the internal validation set was used to tune hyper-parameters to optimize the model, external validation set use for validating the robustness of the screening method to different data.

### Tissue specimens

Tumor tissues were obtained from 8 patients underwent resection of NSCLC (mean age of 54.4 ± 2.3 years, six males). The samples were taken during thoracic surgery, all cancer tissues were identified by HE staining. Four patients were stage I and the rest were stage II, all patients with no history of COPD or other respiratory infectious diseases.

### Real-time PCR (RT-PCR)

Total RNA was extracted from tumor tissues using the TRIzol reagent (TaKaRa, Dalian, China). The primer sequences of 15 mRNAs are listed in Table 7. Qualitative and quantitative analysis of total RNA were using Nanodrop. RNA was reverse transcripted to cDNA and all samples carried out in triplicate and run RT-PCR on an ABI/PRISM 7500 according to the reagent manufacturer's instructions. RT-PCR was performed by SYBR Premix Ex TaqTM II (TaKaRa, Dalian, China).

**Table 7:** The primer sequences of 15 mRNAs.

Gene	Primer sequence (5′–3′)
ADAM10-Forward	TCCACAGCCCATTCAGCAA
ADAM10-Reverse	GCGTCTCATGTGTCCCATTTG
ALDOA-Forward	CGGGAAGAAGGAGAACCTG
ALDOA-Reverse	GACCGCTCGGAGTGTACTTT
CFB-Forward	TCCCTCCTGAAGGCTGGAA
CFB-Reverse	TGTATAGCAAGTCCCGGATCTCA
FGFR1OP-Forward	GAACCGCATCAAGGCTGAACT
FGFR1OP-Reverse	CACTAAACGACCGTCTTTGGTAT
GAPDH-Forward	TGCACCACCAACTGCTTAGC
GAPDH-Reverse	GGCATGGACTGTGGTCATGAG
HMOX1-Forward	AAGATTGCCCAGAAAGCCCTGGAC
HMOX1-Reverse	AACTGTCGCCACCAGAAAGCTGAG
LDHA-Forward	ACCCAGTTTCCACCATGATT
LDHA-Reverse	CCCAAAATGCAAGGAACACT
MESDC2-Forward	CAGAGGTTCATTGTGGGATCAG
MESDC2-Reverse	CTGTCTTGACCGACCAAAAAGT
PKM-Forward	TACCATGCGGAGACCATCAA
PKM-Reverse	AGCAACGGGCCGGTAGAG
PML-Forward	CGCCCTGGATAACGTCTTTTT
PML-Reverse	CTCGCACTCAAAGCACCAGA
PPIA-Forward	ATGGTCAACCCCACCGTGT
PPIA-Reverse	TCTGCTGTCTTTGGGACCTTGTC
TMSB10-Forward	TTTCCTACCCCCGTCCTCTT
TMSB10-Reverse	AGCTTGTGGCTCGTGTCCAT
TUBA1B-Forward	ACCTTAACCGCCTTATTAGCCA
TUBA1B-Reverse	CACCACGGTACAACAGGCA
UBC-Forward	ATTTGGGTCGCGGTTCTTG
UBC-Reverse	TGCCTTGACATTCTCGATGGT
UBE2F-Forward	AGCAAGTAAACTGAAGCGTGAC
UBE2F-Reverse	AACCCTCCGAGTCGAGTCG

### Westernblot

After sufficiently ground and crushed tumor tissue, the protein in tumor tissue is extracted with radioimmunoprecipitation assay (RIPA) buffer, the expression level of 15 mRNAs related protein in cancer tissue were detected by Westernblot. The relevant antibodies used to detect the target protein are as follows: ADAM10 (dilution 1:1000; Abclone, Wuhan, Hubei, China), ALDOA (dilution 1:10,000; Abclone, Wuhan, Hubei, China), CFB (dilution 1: 1000; Abclone, Wuhan, Hubei, China), FGFR1OP (dilution 1: 1000; Abclone, Wuhan, Hubei, China), GAPDH (dilution 1:1000; Abclone, Wuhan, Hubei, China), HMOX1 (dilution 1:1000; Abclone, Wuhan, Hubei, China), LDHA (dilution 1:1000; Abclone, Wuhan, Hubei, China), MESDC2 (dilution 1:1000; Abclone, Wuhan, Hubei, China), PKM (dilution 1:1000; Abclone, Wuhan, Hubei, China), PML (dilution 1:1000; Abclone, Wuhan, Hubei, China), PPIA (dilution 1:1000; Abclone, Wuhan, Hubei, China), TMSB10 (dilution 1:1000; Sigma-Aldrich Chemicals, St. Louis, MO, USA)), TUBA1B (dilution 1:1000; Abclone, Wuhan, Hubei, China), UBC (dilution 1:1000; Abclone, Wuhan, Hubei, China), UBE2F (dilution 1:1000; Abclone, Wuhan, Hubei, China), Beta Actin (dilution 1:1000; Abclone, Wuhan, Hubei, China).

### Statistical analysis

The “survival” package of R software (version 3.5.1) was used to perform survival analysis. Univariate Cox regression analysis was used to evaluate the association between the expression level of mRNA, NSCLC patients’ RFS and patients’survival state in the training set. mRNA expression was considered to be significantly different when the *P*-value was < 0.05, and multivariate Cox regression analysis of highly expressed mRNAs was used to calculate their risk score regression coefficients in the training set ^50–52^. The median value of risk scores in the training set was used as the cutoff point, and NSCLC patients in the training, internal validation, and external validation sets were classified as low risk or high risk corresponding to the cutoff. The Kaplan–Meier estimator and log-rank test were used to assess survival differences between the two groups. Multivariate Cox regression analysis was used to compare the efficacy of the risk score system and the efficacy of clinical characteristics such as stage, age, and sex. ROC curves were used to show the predictive value of RFS in the combined model (risk score combined with stage), risk score model and stage alone. To generate the ROC curves, patients with NSCLC who had a duration of less than 5 years of RFS were excluded if they did not relapse at the last follow-up. We referred to the previous method, set 60 months as the cutoff value of RFS for reasearch the 5-year survival rates, and the remaining NSCLC patients were divided into two groups by this cutoff value ^53,54^. The “pROC” package of R software was used to generate the ROC curve of RFS. Differences observed in the log-rank test, Cox regression analysis, and ROC analysis were considered to be significant if their *P*-values were < 0.05.

Results of RT-PCR and Westernblot are presented as means ± SD. Statistical analyses were calculated via SPSS (version 16.0.0; SPSS, Chicago, IL, USA). One-way ANOVA, Bonferroni post hoc correction (α = 0.0167), and Tukey test were conducted to evaluate significant differences in the data. Statistical significance was set at P < 0.05.

### Functional enrichment analysis

Gene ontology (GO) and Kyoto Encyclopedia of Genes and Genomes (KEGG) analyses were based on the GeneCodis web tool (http://genecodis.cnb.csic.es/) and KOBAS web tool ( http://kobas.cbi.pku.edu.cn/kobas3/?t=1) for functional enrichment analysis of these fifteen-mRNA signature. GO and KEGG category enrichments analyses had cutoff thresholds of *P*-value < 0.05. R software (version 3.5.1) was used to display significant enrichment results in graphical format.

### Protein–protein interaction analysis and mRNA expression validation

STRING (https://string-db.org/) online software was used to analyse the interaction between proteins of fifteen mRNAs and to screen key genes. Gene Expression Profiling Interactive Analysis (GEPIA, http://gepia.cancer-pku.cn/) online software was used to verify the expression of fifteen-mRNA in stage I and II patients with lung adenocarcinoma and lung squamous cell carcinoma.

### Ethics approval and consent to participate

All samples were obtained with informed consent and all protocols were approved by the First Affiliated Hospital of Guangxi Medical University (Scientific and Research Ethics Committee, No. 2020(KY-E-142)). And written informed consent was obtained from all patients participated in our research. This study follows the ethical guidelines of the Declaration of Helsinki 1975.

## Supplementary Information

Supplementary Information.

## Data Availability

All data generated or analyzed during this study are included in this published article.

## Abbreviations

NSCLC: Non-small cell lung cancer
GEO: Gene expression omnibus
ROC: Receiver operating characteristic
GO: Gene ontology
KEGG: Kyoto encyclopedia of genes and genomes
GEPIA: Gene expression profiling interactive analysis
BP: Biological process
CC: Cell component
MF: Molecular function

## References

[1] Chen HY (2007). A five-gene signature and clinical outcome in non-small-cell lung cancer. N. Engl. J. Med..

[2] Cho WC (2016). Application of proteomics in non-small-cell lung cancer. Expert Rev. Proteom..

[3] Howington JA, Blum MG, Chang AC, Balekian AA, Murthy SC (2013). Treatment of stage I and II non-small cell lung cancer: Diagnosis and management of lung cancer, 3rd ed: American College of Chest Physicians evidence-based clinical practice guidelines. Chest.

[4] Hung JJ (2010). Prognostic factors of postrecurrence survival in completely resected stage I non-small cell lung cancer with distant metastasis. Thorax.

[5] Mansoori B (2019). miR-142-3p as tumor suppressor miRNA in the regulation of tumorigenicity, invasion and migration of human breast cancer by targeting Bach-1 expression. J. Cell. Physiol..

[6] Liu T, Xu Z, Ou D, Liu J, Zhang J (2019). The miR-15a/16 gene cluster in human cancer: a systematic review. J. Cell. Physiol..

[7] Tang H (2013). A 12-gene set predicts survival benefits from adjuvant chemotherapy in non-small cell lung cancer patients. Clin. Cancer Res..

[8] Robles AI (2015). An integrated prognostic classifier for stage I lung adenocarcinoma based on mRNA, microRNA, and DNA methylation biomarkers. J. Thorac. Oncol..

[9] Xie Y (2019). Validation of the 12-gene predictive signature for adjuvant chemotherapy response in lung cancer. Clin. Cancer Res..

[10] Yoneyama T (2018). ADAM10 sheddase activity is a potential lung-cancer biomarker. J. Cancer.

[11] Zhou H (2014). High expression of Toll-like receptor 5 correlates with better prognosis in non-small-cell lung cancer: an anti-tumor effect of TLR5 signaling in non-small cell lung cancer. J. Cancer Res. Clin. Oncol..

[12] Ly D, Zhu CQ, Cabanero M, Tsao MS, Zhang L (2017). Role for high-affinity IgE receptor in prognosis of lung adenocarcinoma patients. Cancer Immunol. Res..

[13] Wang M, Zhu J, Lubman DM, Gao C (2019). Aberrant glycosylation and cancer biomarker discovery: a promising and thorny journey. Clin. Chem. Lab. Med..

[14] Sun R (2019). Metabolic gene NR4A1 as a potential therapeutic target for non-smoking female non-small cell lung cancer patients. Thorac. Cancer.

[15] Wu Y (2019). Identification and characterization of sexual dimorphismlinked gene expression profile in hepatocellular carcinoma. Oncol. Rep..

[16] Wei C (2019). Bioinformatics profiling utilized a nine immune-related long noncoding RNA signature as a prognostic target for pancreatic cancer. J. Cell. Biochem..

[17] Riker AI (2008). The gene expression profiles of primary and metastatic melanoma yields a transition point of tumor progression and metastasis. BMC Med. Genomics.

[18] Hage-Sleiman R (2017). Genomic alterations during p53-dependent apoptosis induced by gamma-irradiation of Molt-4 leukemia cells. PLoS ONE.

[19] Roupret M (2011). European guidelines for the diagnosis and management of upper urinary tract urothelial cell carcinomas: 2011 update. Eur. Urol..

[20] Edge SB, Compton CC (2010). The American Joint Committee on Cancer: the 7th edition of the AJCC cancer staging manual and the future of TNM. Ann. Surg. Oncol..

[21] Hattori A, Takamochi K, Okms S, Suzuki K (2019). New revisions and current in the eighth edition of the TNM classification for non-small cell lung cancer. Jpn. J. Clin. Oncol..

[22] Van Bruwaene S, Costello AJ, Van Poppel H (2016). Prognosis of node-positive bladder cancer in 2016. Minerva Urol. Nefrol..

[23] Galon J (2014). Towards the introduction of the 'Immunoscore' in the classification of malignant tumours. J. Pathol..

[24] Galon J (2012). Cancer classification using the immunoscore: a worldwide task force. J. Transl. Med..

[25] Ghasabi M (2019). MicroRNAs in cancer drug resistance: basic evidence and clinical applications. J. Cell. Physiol..

[26] Tian W, Chen J, He H, Deng Y (2013). MicroRNAs and drug resistance of breast cancer: basic evidence and clinical applications. Clin. Transl. Oncol..

[27] Brea-Calvo G, Rodriguez-Hernandez A, Fernandez-Ayala DJ, Navas P, Sanchez-Alcazar JA (2006). Chemotherapy induces an increase in coenzyme Q10 levels in cancer cell lines. Free Radic. Biol. Med..

[28] Jiang Z, Woda BA, Yang XMJ (2002). alpha-Methylacyl coenzyme A racemase as a marker for prostate cancer. JAMA-J. Am. Med. Assoc..

[29] Qian Y, Wang X, Li Y, Cao Y, Chen X (2016). Extracellular ATP a new player in cancer metabolism: NSCLC cells internalize ATP in vitro and in vivo using multiple endocytic mechanisms. Mol. Cancer Res. MCR.

[30] Qian Y (2014). Extracellular ATP is internalized by macropinocytosis and induces intracellular ATP increase and drug resistance in cancer cells. Cancer Lett..

[31] Akram M (2013). Mini-review on glycolysis and cancer. J. Cancer Educ..

[32] Mooring SR, Wang B (2011). HIF-1 inhibitors as anti-cancer therapy. Sci. China Chem..

[33] Semenza GL (2002). HIF-1 and tumor progression: pathophysiology and therapeutics. Trends Mol. Med..

[34] Ye XY, Sun YJ, Xu YH, Chen ZW, Lu S (2016). Integrated In silico-in vitro discovery of lung cancer-related tumor pyruvate kinase M2 (PKM2) inhibitors. Med. Chem..

[35] Yuan S (2016). Knockdown of the M2 isoform of pyruvate kinase (PKM2) with shRNA enhances the effect of docetaxel in human NSCLC cell lines in vitro. Yonsei Med. J..

[36] Danner BC (2010). Long-term survival is linked to serum LDH and partly to tumour LDH-5 in NSCLC. Anticancer Res..

[37] Nair VS, Gevaert O, Davidzon G, Plevritis SK, West R (2014). NF-kappaB protein expression associates with (18)F-FDG PET tumor uptake in non-small cell lung cancer: a radiogenomics validation study to understand tumor metabolism. Lung Cancer.

[38] Nitti M (2017). HO-1 induction in cancer progression: a matter of cell adaptation. Antioxidants.

[39] Mano Y (2007). Fibroblast growth factor receptor 1 oncogene partner as a novel prognostic biomarker and therapeutic target for lung cancer. Cancer Sci..

[40] Guo J (2012). ADAM10 overexpression in human non-small cell lung cancer correlates with cell migration and invasion through the activation of the Notch1 signaling pathway. Oncol. Rep..

[41] Fu H (2018). Aldolase A promotes proliferation and G1/S transition via the EGFR/MAPK pathway in non-small cell lung cancer. Cancer Commun..

[42] Zhang F (2017). Elevated transcriptional levels of aldolase A (ALDOA) associates with cell cycle-related genes in patients with NSCLC and several solid tumors. BioData Min..

[43] Cuezva JM (2004). The bioenergetic signature of lung adenocarcinomas is a molecular marker of cancer diagnosis and prognosis. Carcinogenesis.

[44] Puzone R (2013). Glyceraldehyde-3-phosphate dehydrogenase gene over expression correlates with poor prognosis in non small cell lung cancer patients. Mol. Cancer.

[45] Tang Y (2015). Downregulation of ubiquitin inhibits the proliferation and radioresistance of non-small cell lung cancer cells in vitro and in vivo. Sci. Rep..

[46] Zhou W (2017). Neddylation E2 UBE2F promotes the survival of lung cancer cells by activating CRL5 to degrade NOXA via the K11 linkage. Clin. Cancer Res..

[47] Riihila P (2017). Complement component C3 and complement factor B promote growth of cutaneous squamous cell carcinoma. Am. J. Pathol..

[48] Song C, Su Z, Guo J (2019). Biosci. Rep..

[49] Xiao R (2019). TMSB10 promotes migration and invasion of cancer cells and is a novel prognostic marker for renal cell carcinoma. Int. J. Clin. Exp. Pathol..

[50] Hu Z (2010). Serum microRNA signatures identified in a genome-wide serum microRNA expression profiling predict survival of non-small-cell lung cancer. J. Clin. Oncol..

[51] Sun G (2019). Identification of a five-gene signature with prognostic value in colorectal cancer. J. Cell. Physiol..

[52] Wu YS (2019). A four-miRNA signature as a novel biomarker for predicting survival in endometrial cancer. Gene.

[53] Kang J, D'Andrea AD, Kozono D (2012). A DNA repair pathway-focused score for prediction of outcomes in ovarian cancer treated with platinum-based chemotherapy. J. Natl. Cancer Inst..

[54] Xu G, Zhou Y, Zhou F (2018). Development and validation of an immunity-related classifier of nine chemokines for predicting recurrence in stage I-III patients with colorectal cancer after operation. Cancer Manag. Res..

