# Supplementary material for: A highly expressed mRNA signature for predicting survival in patients with stage I/II non-small-cell lung cancer after operation
Source: Sci Rep. 2021 Mar 12;11:5855. doi: 10.1038/s41598-021-85246-x (PMC7955117; doi:10.1038/s41598-021-85246-x)
Supplement: Supplementary file 1 — Supplementary Information. [file 41598_2021_85246_MOESM1_ESM.pdf]

Supplementary File

Title page

A highly expressed mRNA signature for predicting survival in patients with stage I/II non-small-cell lung cancer after operation

NAN MA[1], LU SI[1], MEILING YANG[1], MEIHUA LI[1*], ZHIYI HE[1*]

Affiliation:

1: Department of Respiratory Medicine, The First Affiliated Hospital of GuangXi Medical University, Nanning, GuangXi 530021, P.R. China

Address of author:

NAN MA, LU SI, MEILING YANG, MEIHUA LI, ZHIYI HE

First Affiliated Hospital of Guangxi Medical University, Nanning, Guangxi 530021, China

E-mail: NAN MA: 66124379@qq.com; LU SI: 564252173@qq.com; MEIHUA LI: li0771@sina.com;

Address for correspondence:

ZHIYI HE, MD, PhD; MEIHUA LI, MD, PhD

Department of Respiratory Medicine, First Affiliated Hospital of Guangxi Medical University, Nanning, Guangxi 530021, China

E-mail: ZHIYI HE : zhiyi-river@163.com;

Tel.: 0771-5356702

* ZHIYI HE and MEIHUA LI contributed equally to this work.

Keywords: classifiter; Non-small cell lung cancer; mRNA; survival analysis; risk classification; microarray

Running title: A multi-RNA classifier for predicting NSCLC recurrence

Original gels of Westernblot

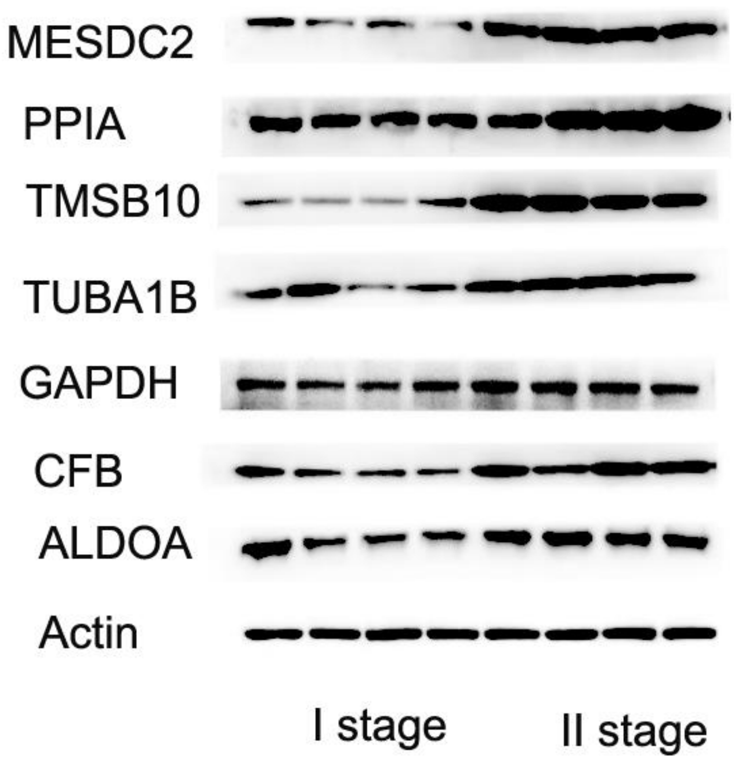

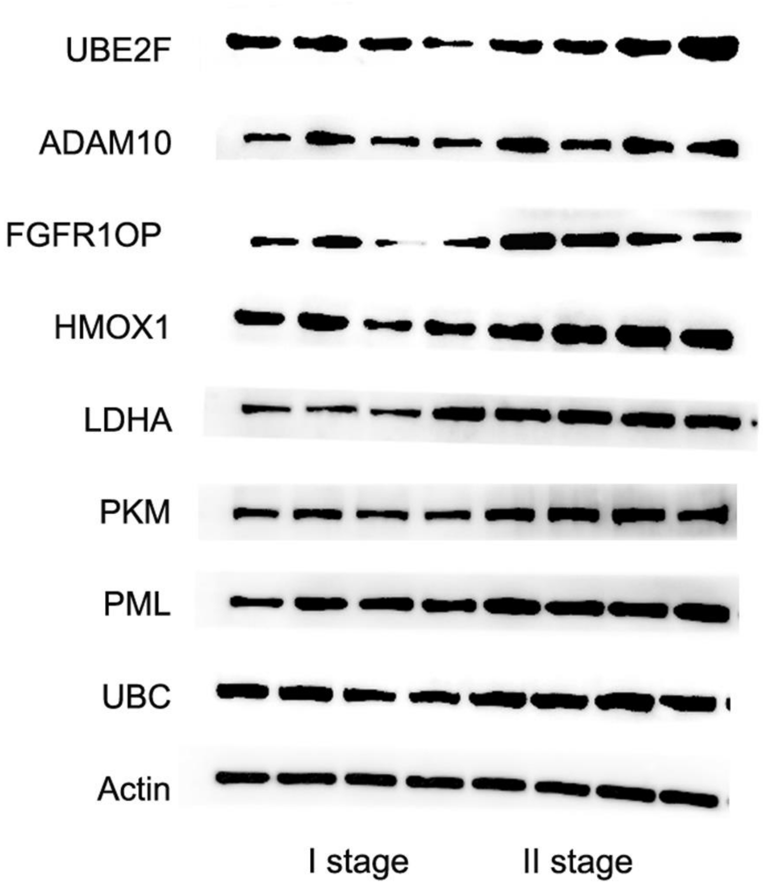
UBE2F
ADAM10
FGFR1OP
HMOX1
LDHA
PKM
PML
UBC
Actin
I stage
II stage

The parameters of our westernblot experiment are as follows: After using the HRP secondary antibody combined with the primary antibody, add ECL luminescent solution in the dark, and scan the protein blots on the membrane scanner. We think the original images of blots previously provided should have been a full-length blots. Details of blots as follow:

1.Original image made by scanner (take blots of MESDC2 as an example):

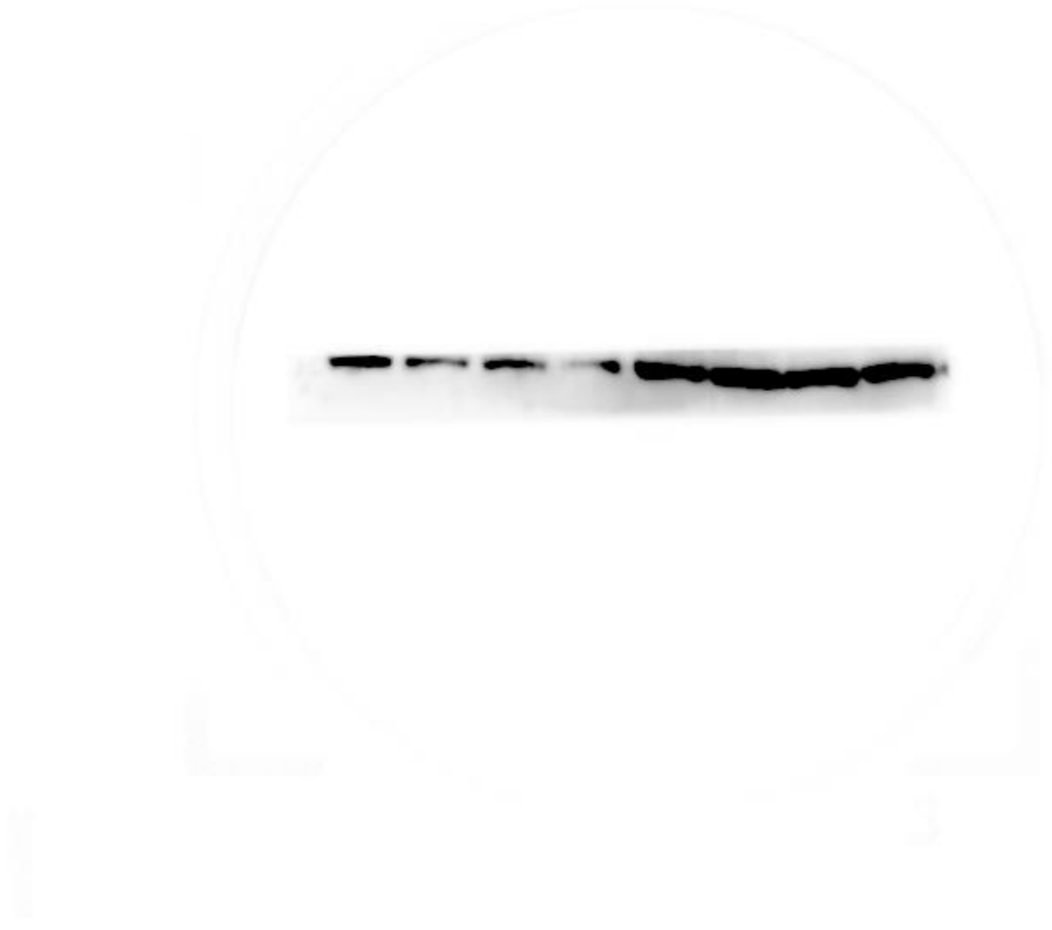

2.The full-length blots cutting by Photoshop software from original image, and place the full-length blots on white background：

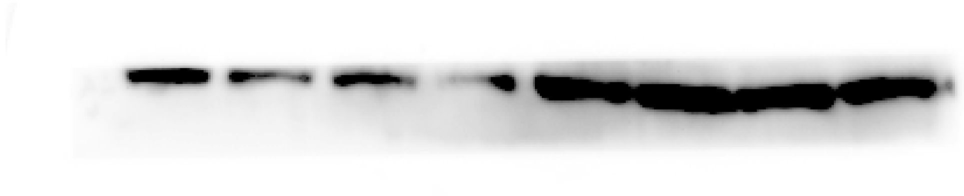

3. The original images of full-length blots we previously provided:

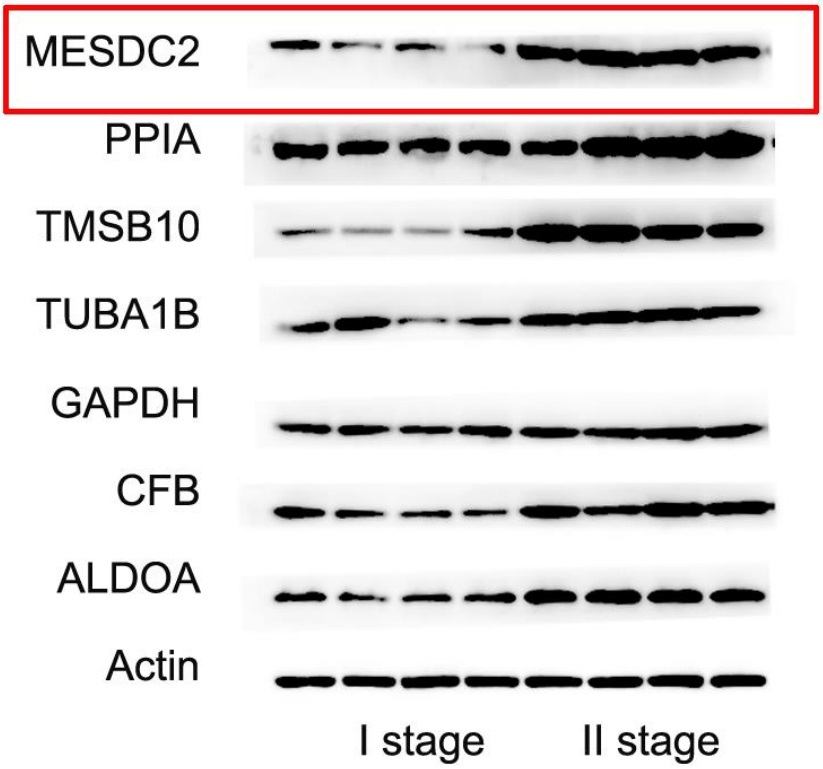

HE staining of NSCLC tumor tissue

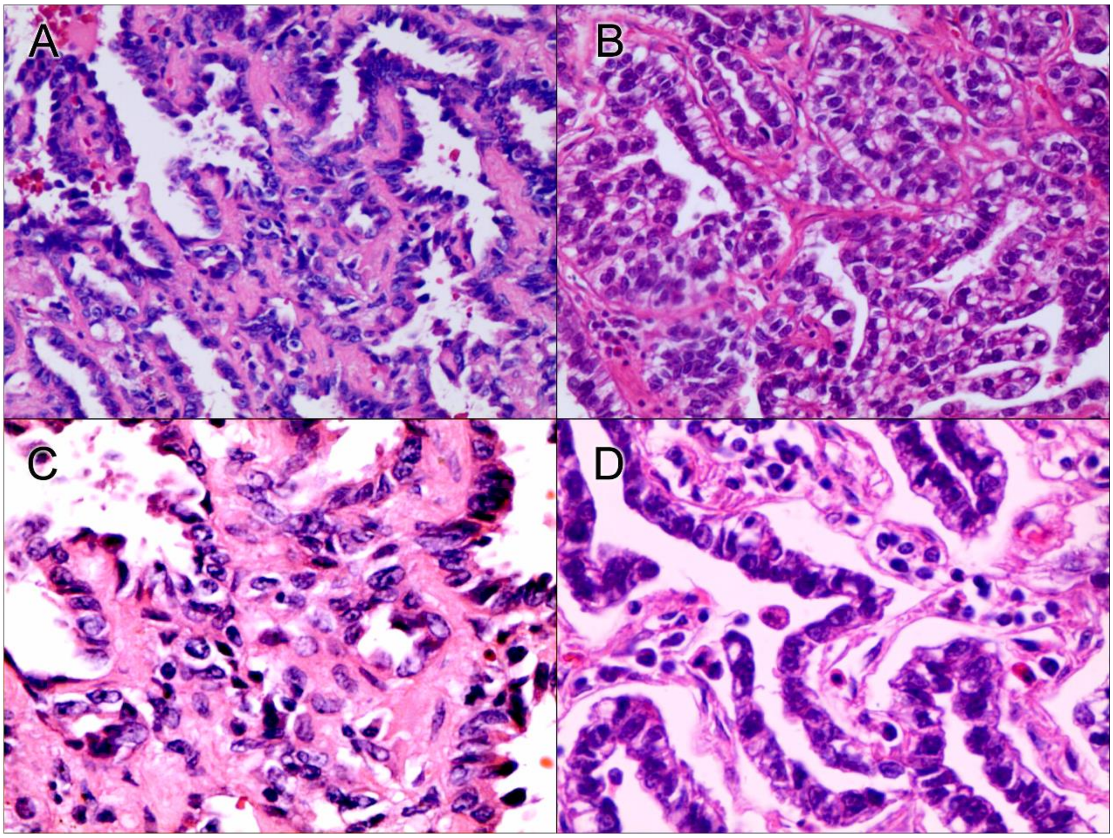

HE staining of NSCLC tumor tissue: A:I stage,200X; B:II stage,200X; C:I stage,400X; D:II stage,400X .
